# Outcomes and Economic Benefits of Penn State Extension’s Dining With Diabetes Program

**DOI:** 10.5888/pcd15.170407

**Published:** 2018-05-03

**Authors:** Debra Griffie, Lynn James, Stephan Goetz, Brandon Balotti, Yau-Huo Shr, Marilyn Corbin, Timothy W. Kelsey

**Affiliations:** 1 College of Agricultural Science, The Pennsylvania State University, University Park, Pennsylvania; 2 Center for Agricultural and Rural Development, Iowa State University, Ames, Iowa

## Abstract

**Introduction:**

Many diabetes education programs address the problem of diabetes, but little attention is given to the economic impact of such programs. Our objective was to assess the effectiveness of a community-based education program in improving diabetes-related lifestyle behaviors and biomarkers and ascertain the economic benefits of the program for adults aged 18 years or older with type 2 diabetes, prediabetes, or no diagnosis of diabetes in Pennsylvania.

**Methods:**

From October 2012 through June 2015, Pennsylvania State University Extension’s Dining with Diabetes program collected data on 2,738 adults with type 2 diabetes or prediabetes and adult family members without diabetes. The program consisted of 4 weekly 2-hour classes and a follow-up class conducted 3 months after the fourth 2-hour class. In the initial class and the follow-up class, participants completed a lifestyle questionnaire and their hemoglobin A_1c_ and blood pressure were measured. Economic benefit was calculated as the medical expenditure cost savings resulting from program participation.

**Results:**

At 3-month follow-up, a significant number of participants had improved their lifestyle behaviors (diet and physical activity), had reductions in hemoglobin A_1c_ and blood pressure, and improved their diabetes status. The Dining with Diabetes program had a 5-year benefit–cost ratio of 2.49 to 3.35.

**Conclusion:**

Participants who completed the Dining with Diabetes program had significant improvements in diabetes-related biomarkers and lifestyle behaviors. If the Dining with Diabetes program were extended to half of the 1.3 million people living with diabetes in Pennsylvania and if they had similar improvements, the 1-year benefit to the state would be approximately $195 million, assuming a conservative 15% decrease in direct medical costs.

## Introduction

Diabetes, the seventh leading cause of death in the United States, entails many complications, and the growing economic costs of the condition are well documented (1). In 2012, the estimated cost of diagnosed diabetes in the United States was $245 billion: $176 billion in direct medical costs and $69 billion in reduced productivity. People with diagnosed diabetes spent an average of $13,700 annually on medical costs, or 2.3 times more than people without diabetes (2). The employed population of people with diabetes also had more absenteeism ($5 billion in indirect costs) and lost productivity ($20.8 billion in indirect costs). Additional costs for people not in the labor force include lost productivity ($2.7 billion), inability to work ($21.6 billion), and early mortality ($18.5 billion) (2).

Many research studies have evaluated education programs that address diabetes, focusing solely on diabetes-related medical costs (1,3). Research also shows that community-based group education programs for diabetes are as effective as individual education and less costly to deliver (4). Research on the economic impact of diabetes education programs is limited.

The primary objective of this research was to assess the effectiveness of a community-based education program, Pennsylvania State University (Penn State) Extension’s Dining with Diabetes (DWD), on improving diabetes-related lifestyle behaviors and biomarkers among adults with type 2 diabetes or prediabetes and among their participating adult family members, most of whom did not have a diagnosis of diabetes but were at risk. The secondary objective was to ascertain the economic benefits of the program. The main hypothesis was that community-based diabetes education would result in positive changes in lifestyle behaviors and biomarkers, thereby reducing diabetes-related medical costs.

## Methods

DWD was a quasi-experimental study conducted in community settings by Penn State Extension from October 2012 through June 2015. We compared baseline and follow-up data for participants by using frequency distributions and paired *t* tests. Study methods were approved by the Penn State institutional review board.

### The DWD program

The DWD program was taught by Extension educators trained in program delivery. These educators were registered dietitians, family consumer science educators, or registered nurses, or held a master’s degree in public health. Educators implemented the evidence-based program in various community settings such as senior centers, churches, libraries, and fire halls throughout the state. The program, based on the social cognitive theory, consisted of 4 weekly 2-hour in-depth classes and a follow-up class conducted 3 months after the fourth in-depth class. Education focused on diabetes-related test numbers, the Idaho Plate Method (5), reduction of saturated fat and sodium, carbohydrate counting, physical activity, and behavior modification. Although the program encourages participants to use their diabetes-related medication consistently, the program does not provide any formal education on medications or medication adjustment. Participants were involved in hands-on food preparation, food tastings, and physical activity. This free program was open to anyone, regardless of income or diabetes status. These characteristics make the program unique, compared with traditional individual and small-group medically based programs.

At the initial class and 3-month follow-up class, participants completed a lifestyle questionnaire, which although not statistically tested, was peer reviewed and refined based on field testing (Appendix). During these classes, we also measured 2 biomarkers for each participant: glycated hemoglobin A_1c_ (HbA_1c_), by using an Afinion AS1000 multi-assay analyzer (Alere), and blood pressure, by using an Omron electronic wrist cuff (model PB629) or Omron arm cuff (model HEM-712CLC) (Omron Healthcare, Inc). We assessed blood pressure because of the interrelationships among blood pressure, heart disease, and diabetes. A 10-point drop in blood pressure reduces the risk of stroke by 27% and the risk of heart attack by 12%; for people with type 2 diabetes, it reduces the overall risk of heart disease by 12% (6).

Participants were recruited from 46 of 67 counties in Pennsylvania (62% rural and 38% urban, as defined by the Center for Rural Pennsylvania [7]) by the following means: word of mouth (21%), promotional brochures (12%), newspaper advertisements (42%), referrals from health care providers (4%) and other (24%). From October 2012 through June 2015, we collected baseline data from 2,738 participants and 3-month follow-up data from 1,936 participants. The attrition rate was 29% (802 of 2,738). We defined “completers” as people who completed the biomarker testing and lifestyle questionnaire at both baseline and follow-up.

Persons aged 18 or older with type 2 diabetes, prediabetes, or no prior diagnosis of diabetes (usually a family member or friend), were invited to attend the program. Because the program was funded in part by the Centers for Medicare & Medicaid Services, most participants were eligible for Medicare or Medicaid. The median age of participants was 68 years, and 68% were aged 65 or older, making them Medicare eligible. Just over 26% of participants met the income eligibility requirements for Medicaid, and 15% received Medicaid insurance coverage. Most participants were white (88%), followed by African American (7.2%), Hispanic/Latino (2.7%), American Indian or Alaska Native (1.9%), Asian (0.7%), and other (1.2%). Most participants were women (74.1%) and had at least a high school education or GED (40.7%). Completers were significantly more likely to be older by 2 years (mean, 68 vs 66), to be white (5%), to have an income of more than $25,000 (6%), to be from rural areas (7%), and to have social support (5%). Family members, regardless of diabetes status, were encouraged to attend the program to offer social support and reduce personal diabetes risk.

### Analysis of biomarkers and questionnaires

To assess the effectiveness of the DWD program, we analyzed data on biomarkers and data from the lifestyle questionnaires. We analyzed only data from completers. First, we conducted a descriptive analysis of their 1) self-reported knowledge, attitudes, and behaviors and 2) HbA_1c_ and blood pressure measurements. For the latter analysis, we categorized participants by their diabetes status at baseline: no diabetes (HbA_1c_ <5.7), prediabetes (HbA_1c_ 5.7–6.4), controlled diabetes (HbA_1c_ 6.5–7.0), and uncontrolled diabetes (HbA_1c_ >7.0). We calculated frequency distributions and compared before-and-after results. To test differences in before-and-after results, we used paired *t* tests for continuous values, the Wilcoxon signed rank test for ordinal categorical variables, and the McNemar test for dichotomous categorical variables. Next, we determined the number of participants with uncontrolled diabetes at baseline who changed their diabetes status at 3-month follow-up. We also determined the number of participants in each diabetes category at baseline and follow-up and assessed the percentage of participants who moved from one diabetes status to another. We considered a *P* value of ≤.05 to be significant. We used SPSS version 22 (IBM Corp) for all analyses of biomarkers and survey questions.

### Cost-benefit analysis

Our cost-benefit analysis consisted of calculating 1) the annual direct and indirect economic costs associated with diabetes and prediabetes, 2) the medical cost savings of the DWD program, 3) the estimated costs of delivering the DWD program, 4) the 5-year and 10-year benefit-costs ratios, and 5) the 1-year benefit to the state of implementing the DWD program to half of the estimated number of people living with diabetes in Pennsylvania. We calculated the annual direct and indirect economic costs associated with diabetes and prediabetes for the 2,249 participants in the DWD program with known diabetes status at baseline. We estimated total economic costs as the sum of direct medical costs and indirect costs, wherein we assumed, on the basis of previous research, that direct medical costs were 84.8% of total costs (8). Direct medical costs consisted of hospitalization, outpatient care, and outpatient medications and supplies. Indirect costs include work absences, reduced productivity, reduced labor force participation because of disability, and mortality (2).

The general approach of 2 previous studies was used to calculate the medical cost savings of the DWD program (8,9). This approach assumes a partial reduction in annual medical costs for participants who have an HbA_1c_ of 6.4 or greater at baseline and an HbA_1c_ of less than 6.4 at follow-up (8). Previous research found that direct medical costs for patients with diabetes who sustained control (HbA_1c_ <7.0) over 3 years decreased by about 15% (9). We considered only direct cost savings; we did not consider reductions in spending on other diabetes-related complications such as diabetic retinopathy. 

As a comparison, we calculated medical cost savings by using an alternative method: we estimated medical cost savings for a participant with diabetes at baseline who had any reduction in HbA_1c_. We used a gamma regression model to estimate the relationship between glycemic control and medical expenses with controls such as patients’ age and sex and HbA_1c_, heart disease, hypertension, and hyperlipidemia status (10). We included heart disease because approximately one-third of people with diabetes also have heart disease. For people with diabetes and heart disease, medical costs are 3.15 times higher than for people with diabetes alone (2,3). 

Our annual medical cost estimates considered the economic impact of the DWD program only for participants with diabetes or prediabetes who had a reduction in HbA_1c_. We did not consider the impact of DWD on the nonmedical costs of diabetes reduction; this impact has not been considered in the scientific literature. 

Because our study design was quasi-experimental, we also did not consider the potential economic impact of reducing the amount of increase in HbA_1c_ levels for participants who would have had further increases had they not participated in the program. Furthermore, quantifying the cost-saving effects of program participation for people with prediabetes was not feasible because research on cost savings for people who no longer have prediabetes does not exist (9).

To illustrate the sensitivity of our results to alternative assumptions about the medical cost savings associated with the DWD program, we estimated annual cost savings by using both medical costs and indirect cost estimates from a previous study, in which patients with diabetes achieved normal HbA_1c_ levels and 25%, 50%, and 100% of the participants incurred no further expenses (8).

Next, we calculated the estimated program delivery costs of DWD. These costs consist of variable factors that depend on the number of participants in a session and the fixed cost of delivering a session. We calculated 5-year and 10-year benefit–cost ratios, assuming a 15% decrease in direct medical costs as a result of improved diabetes status and then using the more conservative estimates of previous studies (3); to calculate these benefit–cost ratios, we divided the 5-year and 10-year benefits by the costs for a given number of participants. Finally, we calculated the 1-year benefit to the state of implementing the DWD program to half of the 1,348,305 people living with diabetes in Pennsylvania (both diagnosed and undiagnosed), assuming they would have similar reductions in HbA_1c_ to the reductions found among our study population and assuming a 15% decrease in direct medical costs when diabetic status improves (10,11).

## Results

### Lifestyle questionnaire

A significantly greater percentage of participants at 3-month follow-up than at baseline indicated that they could explain their HbA_1c_ (86.3% vs 67.2%) and blood pressure (89.3% vs 79.8%) results somewhat well or very well (Table 1). A significantly greater percentage of participants at follow-up (66.7%) than at baseline (57.6%) indicated that they were confident they could keep their diabetes under control. However, we found no significant change in participants’ adherence to prescribed blood pressure, cholesterol, or blood glucose medications. Most participants at baseline (81.0%) and follow-up (80.9%) were taking their medications as prescribed. Participants significantly increased the number of days per week in which they exercised for 20 minutes or more (from an average of 2.9 days to 3.4 days) and in which they ate a variety of fruits and vegetables (from an average of 5.1 days to 5.4 days). 

**Table 1:** Selected Self-Reported Data on Basic Knowledge, Attitude, and Behavior, Lifestyle Questionnaire Administered by Pennsylvania State University Extension’s Dining With Diabetes Program, October 2012–June 2015^a^

Impact of Program	No. of Respondents	Baseline, % or no.	3-Month Follow-Up, % or no.	*P* Value
**Basic knowledge**				
Can explain your HbA1c result to someone else somewhat well or very well	1,676	67.2%	86.3%	<.001^b^
Can explain your blood pressure result to someone else somewhat well or very well	1,790	79.8%	89.3%	<.001^b^
**Attitude**				
Agree or strongly agree that I feel confident I can keep my diabetes under control	1,448	57.6%	66.7%	<.001^b^
**Consistent use of medications**				
Did not forget to take a pill or injection during the last 7 days	1,608	81.0%	80.9%	.19^c^
Disagree or strongly disagree that sometimes I am careless about taking my medicines	1,713	70.8%	73.4%	.06^b^
**Ability to adopt healthy behaviors**				
Average no. of days per week exercised for 20 min or more	1,702	2.9	3.4	<.001^d^
Average no. of days per week ate a variety of fruits and vegetables	1,717	5.1	5.4	<.001^d^

a Baseline data were collected from 2,738 participants and 3-month follow-up data from 1,936 participants. Data were analyzed only for participants who completed the baseline questionnaire and the 3-month follow-up questionnaire (Appendix). The program consisted of 4 weekly in-depth classes and a follow-up class conducted 3 months after the fourth in-depth class. The baseline questionnaire was administered during the first in-depth class.

b Determined by Wilcoxon signed rank test.

c Determined by McNemar test.

d Determined by paired-samples *t* test.

### Biomarkers

**Figure 1:**
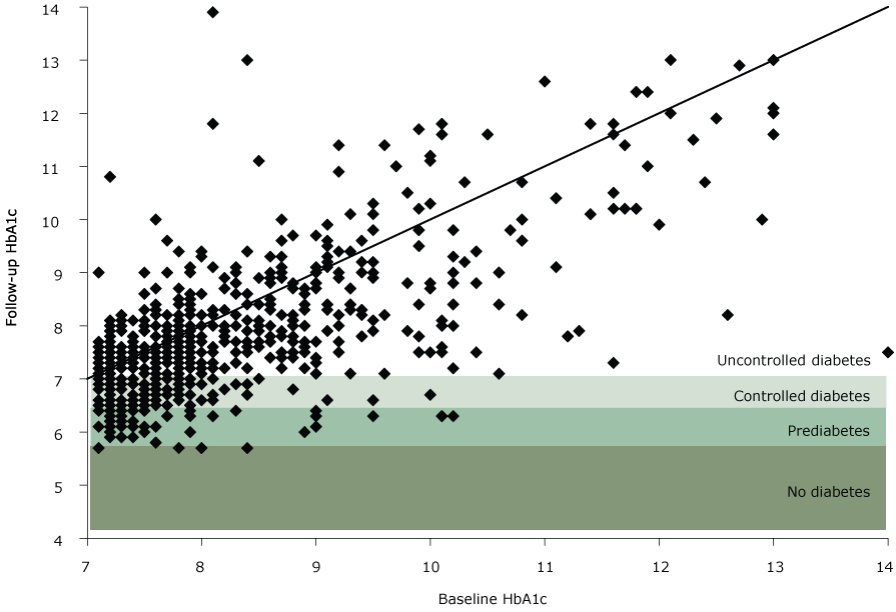
Follow-up HbA_1c_ compared with baseline HbA_1c_ for participants with uncontrolled diabetes at baseline, Pennsylvania State University Extension’s Dining With Diabetes Program, October 2012–June 2015. Each point represents a study participant. Points below the diagonal line indicate a participant with a decrease in HbA_1c_, whereas points above the line indicate a participant with an increase in HbA_1c_. A point in the shaded areas indicates that a decrease was large enough to move the participant into a new, lower category of diabetes. The following categories of diabetes status were used: no diabetes (HbA_1c_ <5.7), prediabetes (HbA_1c_ 5.7–6.4), controlled diabetes (HbA_1c_ 6.5–7.0), and uncontrolled diabetes (HbA_1c_ >7.0).

Program participation was associated with positive effects on participants’ biomarkers. Of the 1,783 participants with baseline and follow-up measurements, 887 (49.7%) had a decrease in HbA_1c_, 175 (9.8%) stayed the same, and 721 (40.4%) had an increase. At follow-up, 368 (20.6%) participants had a decrease in HbA_1c_ large enough to lower their diabetes status. Of the 592 participants who had uncontrolled diabetes at baseline, 102 (17.2%) changed to controlled diabetes and 58 (9.8%) changed to prediabetes at follow-up (Figure 1). These changes translate to a 5.9% decrease in HbA_1c_ for 160 of 592 (27%) participants who had uncontrolled diabetes at baseline. 

**Figure 2:**
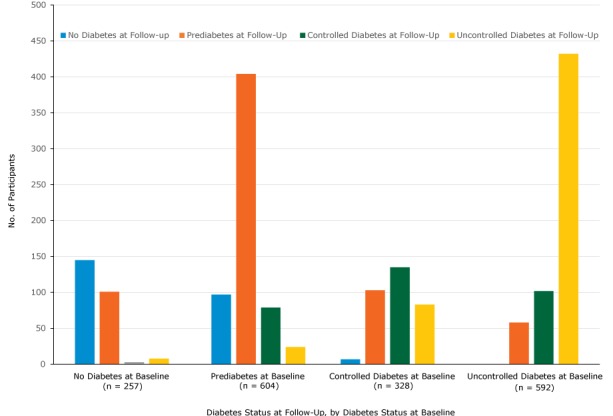
Number of participants in each diabetes category, determined by HbA_1c_ measurements, at baseline and follow-up, Pennsylvania State University Extension’s Dining With Diabetes Program, October 2012–June 2015. The following categories of diabetes status were used: no diabetes (HbA_1c_ <5.7), prediabetes (HbA_1c_ 5.7–6.4), controlled diabetes (HbA_1c_ 6.5–7.0), and uncontrolled diabetes (HbA_1c_ >7.0). **Table:** 

Of the 328 participants who had controlled diabetes at baseline, 103 (31.4%) changed to prediabetes and 8 (2.4%) changed to no diabetes at follow-up. Of the 604 participants who had prediabetes at baseline, 97 (16.1%) changed to no diabetes at follow-up and 404 (66.9%) stayed the same (Figure 2).

Diabetic Status at Baseline per HbA_1c_ Measurement	Baseline	No Diabetes at Follow-up	Prediabetes at Follow-up	Controlled Diabetes at Follow-up	Uncontrolled Diabetes at Follow-up
No diabetes	257	145	101	3	8
Prediabetes	604	97	404	79	24
Controlled diabetes	328	7	103	135	83
Uncontrolled diabetes	592	0	58	102	432

Numerous participants had increases in HbA_1c_ measurements from baseline to follow-up, but we found a significant increase in mean HbA_1c_ in only 1 category, no diabetes at baseline: mean HbA_1c_ increased from 5.3 to 5.7 (Table 2). Of the 257 participants who had no diabetes at baseline, as determined by HbA_1c_ measurements, 62 reported being told they had prediabetes. Some of these 257 participants also reported taking medications (ie, blood glucose pills [n = 38] and insulin [n = 7]).

**Table 2:** Mean Values for Blood Pressure and HbA1c Measurements, Pennsylvania State University Extension’s Dining With Diabetes Program, October 2012–June 2015^a^

Biomarker by Diabetes Status	No. of Participants With Baseline and 3-Month Follow-Up Measurements	Baseline	3-Month Follow-Up	*P* Value^b^
**No diabetes (baseline HbA1c <5.7)**				
Systolic blood pressure, mm Hg	262	135.0	127.9	<.001
Diastolic blood pressure, mm Hg	262	77.8	74.3	<.001
HbA1c	257	5.3	5.7	<.001
**Prediabetes (baseline HbA1c 5.7–6.4)**				
Systolic blood pressure, mm Hg	590	137.2	132.2	<.001
Diastolic blood pressure, mm Hg	590	75.8	74.0	<.001
HbA1c	604	6.1	6.1	.11
**Controlled diabetes (baseline HbA1c 6.5–7.0)**				
Systolic blood pressure, mm Hg	328	138.1	135.2	.005
Diastolic blood pressure, mm Hg	328	75.6	74.8	.16
HbA1c	328	6.7	6.7	.53
**Uncontrolled diabetes (baseline HbA1c >7.0)**				
Systolic blood pressure, mm Hg	588	141.1	136.4	<.001
Diastolic blood pressure, mm Hg	588	76.8	74.1	<.001
HbA1c	592	8.4	7.9	<.001

a Of the 2,738 participants in the Dining with Diabetes program, 1,810 participants had both baseline and follow-up blood pressure measurements and 1,783 participants had both baseline and follow-up HbA1c measurements. Data were analyzed only for participants who completed the baseline questionnaire and the 3-month follow-up questionnaire (Appendix). The program consisted of 4 weekly in-depth classes and a follow-up class conducted 3 months after the fourth in-depth class. The baseline questionnaire was administered during the first in-depth class.

b Determined by paired-samples *t* test.

The DWD program was associated with significant decreases in systolic and diastolic blood pressure measurements across all diabetes categories (Table 2). Of the 1,810 participants with both baseline and follow-up blood pressure measurements, 1,074 participants (59.3%) had a decrease in blood pressure, including 181 of 262 (69.1%) with no diabetes, 359 of 590 (60.8%) with prediabetes, 184 of 328 (56.1%) with controlled diabetes, and 350 of 588 (59.5%) with uncontrolled diabetes. 

### Cost benefit analysis

We estimated the annual direct and indirect economic costs associated with diabetes and prediabetes for Pennsylvania to be $17,589,584. The annual medical cost savings resulting from the DWD program for participants with diabetes (n = 920) was calculated to be $266,022 (Table 3). Assuming that the participants’ diabetes status remained unchanged and the cost savings persisted, we calculated the discounted net present value of these savings at a 3% interest rate to be $1,254,852 over 5 years and $2,337,289 over 10 years. 

**Table 3:** Medical Cost Savings Resulting From Pennsylvania State University Extension’s Dining With Diabetes Program, October 2012–June 2015

Estimation Approach	Medical Cost Savings, $	Indirect Cost Savings, $	Total Cost Savings, $	Covered Participants
15% decrease in direct medical costs when diabetes status improved^a^	$266,022^b^	Not applicable	Not applicable	Participants with diabetes
Decrease in medical costs when HbA1c is reduced^c^	$197,585^d^	Not applicable	Not applicable	Participants with diabetes
Full recovery for 25% of participants with diabetes^a^	$430,039	$72,664	$502,703	Participants with diabetes and prediabetes
Full recovery for 50% of participants with diabetes^a^	$860,078	$145,327	$1,005,405	Participants with diabetes and prediabetes
Full recovery for 100% of participants with diabetes^a^	$1,720,156	$290,655	$2,010,811	Participants with diabetes and prediabetes

a Assumption based on Dall and colleagues (8).

b 5-year savings is $1,254,852 and 10-year savings is $2,337,298.

c Based on Gilmer and colleagues (10).

d 5-year savings calculated to be $933,502 and 10-year savings to be $1,750,975.

The annual medical cost savings for the 533 participants with diabetes at baseline who had any decrease in HbA_1c_ levels during the DWD program was calculated to be $197,585. At a 3% interest rate, medical cost savings for these participants would amount to net present values of $933,502 over 5 years and $1,750,975 over 10 years.

For each participant, the variable cost of program delivery was determined to be $49.25, and the total fixed cost of one session was determined to be $4,293. Assuming an average class size of 12 participants, the calculated total cost per person of delivering the DWD program was $407.

For the 920 participants enrolled in the DWD program who had diabetes at baseline, the total cost of delivering the program was $374,440. This translated to a 5-year benefit-cost ratio of 3.35, and a 10-year ratio of 6.24, assuming a 15% decrease in direct medical costs as a result of improved diabetes status ($1,254,852). Using the more conservative estimates, we found the corresponding 5-year and 10-year benefit cost ratios to be 2.49 and 4.68, respectively. Therefore, our results suggest that in the DWD program, for every dollar spent delivering the program a 5-year benefit between $2.49 and $3.35 was realized. Over a decade, the benefit would range from $4.68 to $6.24. If the DWD program were extended to half of the people with diabetes in Pennsylvania, the 1-year benefit to the state would be approximately $195 million. Given a 3% interest rate, the 5-year and 10-year benefit would be approximately $920 million and $1.71 billion, respectively.

## Discussion

The DWD program in Pennsylvania is a cost-effective community-based program that helped most participants change their behavior and significantly improve their HbA_1c_ and blood pressure. The significant reductions in HbA_1c_ in our study are most likely not attributed to maturation effects, because diabetes does not typically improve without some form of intervention (12). A previous study suggested that diabetes self-management education improved blood glucose control; this study found a reduction in HbA_1c_ of nearly 0.6% compared with normal care alone (13). Another study determined that more than 10 contact hours in diabetes self-management education was associated with a significant reduction in HbA_1c_ (11). Penn State’s DWD program, although shorter in duration (8 contact hours), showed a reduction of 5.9% in HbA_1c_ for 27% of participants with uncontrolled diabetes at baseline. Better control of biomarkers, including an HbA_1c_ of less than 7.0 and controlled blood pressure, reduces long-term medical complications such as heart disease, myocardial infarction, stroke, kidney disease and failure, diabetic neuropathy and amputations, eye disease, blindness, and early mortality. The lifestyle modification focus of the DWD program assisted participants in significantly increasing their intake of fruits and vegetables and their physical activity. These positive changes translated into 5-year cost savings that ranged from $2.49 to $3.35 for each dollar spent delivering the program.

The DWD program does not screen participants for weight, but many enrolled in the program do lose weight by following the program’s diet and exercise recommendations. The US Preventive Services Task Force recommends screening for diabetes among all overweight or obese adults aged 40 to 70 (14). Implementation of these recommendations will increase the need for education on lifestyle modification for people who are identified as having prediabetes. With more community and health center programs to address diabetes and prediabetes, major complications can be prevented.

Our study has several limitations. First, the research was limited to an evaluation of the health and economic outcomes of an educational program, and further research is warranted to compare health outcomes in an intervention group with health outcomes in a control group. Second, DWD educators in Pennsylvania are distributed across the state, and most are in rural areas where most of the population is white. Because of staffing limitations, our study underrepresented racial/ethnic minority groups. Third, we found discrepancies between self-reported data and objectively measured data. For example, 257 participants reported they did not have diabetes at baseline. Their HbA_1c_ measurement showed they did not have diabetes, but they also reported taking diabetes medications; these participants would have been categorized incorrectly as having no diabetes, when they should have been categorized as having prediabetes or diabetes. These discrepancies may explain the rise in HbA_1c_ among some participants at follow-up. Finally, our research targeted Medicare/Medicaid–eligible individuals; research on reaching young adults (aged <30) and teenagers with diabetes is needed.

Future program adaptations being considered include offering flexible class times for working audiences and offering more activities as part of a company’s worksite wellness program. An online version of DWD was created to deliver the program to adults aged 18 to 60 who may not be able to attend the face-to-face program during working hours. As the incidence of prediabetes continues to rise, integrating the DWD program into schools and college settings warrants further investigation. More studies are needed to investigate the indirect (nonmedical) benefits of controlled diabetes, such as increased attendance at work or school and increased productivity.

Our questionnaire was revised several times to improve lesson content and teaching strategies. It needs to be further revised to count the number of participants with heart disease, hyperlipidemia, kidney disease, and diabetic neuropathy and how these conditions relate to employment or activities of daily living for better economic analysis.

DWD is offered as a national Extension program, but the national program has not undergone any type of economic analysis. The national curriculum is similar to the curriculum in Pennsylvania but does not test biomarkers; biomarker testing of HbA_1c_ is more accurate than self-report of HbA_1c_. With the addition of biomarker testing, Extension programs in other states should be able to show similar reductions in HbA_1c_ and have similar economic outcomes with similar demographic groups.

## Appendix. Lifestyle Questionnaires

### A. Questionnaire Administered During Initial Class

**1.** How did you hear about this program? (please check all that apply)

□ Word of mouth □ Promotional brochure □ Newspaper advertisement □ Referral from healthcare provider □ Other:

**2.** Are you here today . . . ?

□ By yourself □ With a spouse □ With another relative □ With a friend □ With someone you take care of □ Other:

**3.** Have you been told you have diabetes? □ Yes □ No □ Not sure

**4.** Have you been told you have prediabetes or are at high risk for developing diabetes? **□** Yes **□** No **□** Not sure

**5.** If you have diabetes, for how many *years* have you had diabetes? **□** Less than 5 **□** 5–10 **□** More than 10

**6.** Do you take pills for high blood glucose (blood sugar)? □ Yes □ No □ Not sure

**7.** Do you take insulin? □ Yes □ No □ Not sure

**8.** If you take insulin, for how many *years* have you taken it? □ Less than 5 □ 5–10 □ More than 10

**9.** How would you describe your general health? □ Good □ Fair □ Poor

**10.** Approximately how many *months *ago did you last see a healthcare provider? □ Less than 6 □ 6–12 □ More than 12 □ Never

**11.** Do you have medicine(s) for high blood pressure, high cholesterol, or high blood glucose (sugar)? □ Yes □ No □ Not sure

**12.** During the last 7 days, did you skip or forget to take at least one of these pills or an injection? □ Yes □ No □ Not sure □ I don’t take these medicines

**13.** Sometimes I am careless about taking my medicines. □ Strongly agree □ Agree □ Neither agree nor disagree □ Disagree □ Strongly disagree

**14.** How well do you think you could explain your *A1C* result to someone else? □ Very well □ Somewhat well □ Not at all well

**15.** How well do you think you could explain your *blood pressure* result to someone else? □ Very well □ Somewhat well □ Not at all well

**Table:**

16. Do you agree or disagree with the following statements?	Strongly Agree	Agree	Neither Agree or Disagree	Disagree	Strongly Disagree
Diabetes is not that serious, especially if you feel fine					
I feel confident that I can keep my diabetes under control					

**Table:**

17. During the last month, how much of a problem was each of these for you?	Not at All	Slight	Moderate	Somewhat Serious	Serious	Very Serious
Feeling overwhelmed by the demands of living with diabetes						
Feeling that I am often failing with my diabetes routine						

**Table:**

18. On how many of the last 7 days did you . . . ?	None	1	2	3	4	6	Every Day
Exercise for 20 minutes or more?							
Eat a variety of fruits and vegetables?							
Sleep between 6.5 and 8.5 hours at night?							

**19.** Gender: □ Male □ Female

**20.** Date of birth: __/__/____

**21.** Age:

**22.** Health insurance (please check all that apply) **□** Private **□** Medicaid **□** Medicare (fee for service) **□** Medicare (advantage) **□** None

**23.** Do you smoke? **□** I currently smoke **□** I used to smoke but I quit **□** I never smoked

**24.** Are you pregnant? □ Yes □ No □ Not sure

**25.** How do you describe yourself? (please check all that apply) □ Asian □ Black or African-American □ Hispanic/Latino □ Native American or Alaska Native □ Native Hawaiian or Other Pacific Islander □ White/Caucasian □ Other:

**26.** What is your approximate annual household income? □ Less than $15,000 □ $15,000–$24,999 □ $25,000–$49,999 □ $50,000–$74,999 □ $75,000 or more

**27.** Highest education level completed: □ Less than high school graduate □ High school graduate/GED □ Some college or trade school □ College graduate or higher

### B. Questionnaire Administered 3 Months After Program End

**1.** Have you been told you have diabetes? □ Yes □ No □ Not sure

**2.** Have you been told you have pre-diabetes or are at high risk for developing diabetes? □ Yes □ No □ Not sure

**3.** Do you take pills for high blood glucose (blood sugar)? □ Yes □ No □ Not sure

**4.** Do you take insulin? □ Yes □ No □ Not sure

**5.** Have you shown the lab results from this class to a healthcare provider? □ Yes □ No

**6.** Have you made an appointment to see a healthcare provider since the start of the program? □ Yes □ No

**7.** How would you describe your general health? □ Good □ Fair □ Poor

**8.** Do you have medicine(s) for high blood pressure, high cholesterol, or high blood glucose (sugar)? □ Yes □ No □ Not sure

**9.** During the last 7 days, did you skip or forget to take at least one of these pills or an injection? □ Yes □ No □ Not sure □ I don’t take these medicines

**10.** Sometimes I am careless about taking my medicines. □ Strongly agree □ Agree □ Neither agree nor disagree □ Disagree □ Strongly disagree

**11.** How well do you think you could explain your *A1C* result to someone else? □ Very well □ Somewhat well □ Not at all well

**12.** How well do you think you could explain your *blood pressure* result to someone else? **□** Very well □ Somewhat well □ Not at all well

**Table:**

13. Do you agree or disagree with the following statements?	Strongly Agree	Agree	Neither Agree nor Disagree	Disagree	Strongly Disagree
Diabetes is not that serious, especially if you feel fine					
I feel confident that I can keep my diabetes under control					

**Table:**

14. During the last month, how much of a problem was each of these for you?	Not at All	Slight	Moderate	Somewhat Serious	Serious	Very Serious
Feeling overwhelmed by the demands of living with diabetes						
Feeling that I am often failing with my diabetes routine						

**Table:**

15. On how many of the last 7 days did you . . . ?	None	1	2	3	4	5	6	Every Day
Exercise for 20 minutes or more?								
Eat a variety of fruits and vegetables?								
Sleep between 6.5 and 8.5 hours at night?								

**16.** Do you smoke? **□** I currently smoke □ I used to smoke but I quit □ I never smoked

**17.** Are you pregnant? □ Yes □ No □ Not sure
